# Implementing a ward-based programme to improve care for older inpatients: process evaluation of the cluster randomised CHERISH trial

**DOI:** 10.1186/s12913-023-09659-2

**Published:** 2023-06-21

**Authors:** Alison M. Mudge, Prue McRae, Adrienne Young, Irene Blackberry, Karen Lee-Steere, Sally Barrimore, Tara Quirke, Gillian Harvey

**Affiliations:** 1 Royal Brisbane and Women’s Hospital Department of Internal Medicine and Aged Care, Herston, Australia; 2 Queensland University of Technology Institute of Health and Biomedical Innovation, Kelvin Grove, Australia; 3 University of Queensland Faculty of Medicine, Brisbane, Australia; 4 Royal Brisbane and Women’s Hospital Department of Nutrition and Dietetics, Herston, Australia; 5 LaTrobe University John Richards Centre for Rural Ageing Research, Wodonga, Australia; 6 University of Queensland Faculty of Health and Behavioural Sciences, Brisbane, Australia; 7 The Prince Charles Hospital, Chermside, Australia; 8 Consumer Advocate Dementia Training Australia, Brisbane, Australia; 9 Flinders University College of Nursing and Health Sciences, Bedford Park, Australia

**Keywords:** Implementation, Evaluation, Delirium, Age-friendly hospitals, Facilitation

## Abstract

**Background:**

Older inpatients are at high risk of hospital-associated complications, particularly delirium and functional decline. These can be mitigated by consistent attention to age-friendly care practices such as early mobility, adequate nutrition and hydration, and meaningful cognitive and social activities. Eat Walk Engage is a ward-based improvement programme theoretically informed by the i-PARIHS framework which significantly reduced delirium in a four-hospital cluster trial. The objective of this process evaluation was to understand how Eat Walk Engage worked across trial sites.

**Methods:**

Prospective multi-method implementation evaluation on medical and surgical wards in four hospitals implementing Eat Walk Engage January 2016-May 2017. Using UK Medical Research Council guidance, this process evaluation assessed context, implementation (core components, implementation strategies and improvements) and mechanisms of impact (practice changes measured through older person interviews, structured mealtime observations and activity mapping) at each site.

**Results:**

The four wards had varied contextual barriers which altered dynamically with time. One ward with complex outer organisational barriers showed poorer implementation and fewer practice changes. Two experienced facilitators supported four novice site facilitators through interactive training and structured reflection as well as data management, networking and organisational influence. Novice site facilitators used many implementation strategies to facilitate 45 discrete improvements at individual, team and system level. Patient interviews (42 before and 38 after implementation) showed better communication about program goals in three sites. Observations of 283 meals before and 297 after implementation showed improvements in mealtime positioning and assistance in all sites. Activity mapping in 85 patients before and 111 patients after implementation showed improvements in cognitive and social engagement in three sites, but inconsistent changes in mobility. The improvements in mealtime care and cognitive and social engagement are plausible mediators of reduced delirium observed in the trial. The lack of consistent mobility improvements may explain why the trial did not show reduction in functional decline.

**Conclusions:**

A multi-level enabling facilitation approach supported adaptive implementation to varied contexts to support mechanisms of impact which partly achieved the programme goals. Contexts changed over time, suggesting the need for adequate time and continued facilitation to embed, enhance and sustain age-friendly practices on acute care wards and optimise outcomes.

**Trial registration:**

The CHERISH trial was prospectively registered with the ANZCTR (http://www.anzctr.org.au): ACTRN12615000879561.

**Supplementary Information:**

The online version contains supplementary material available at 10.1186/s12913-023-09659-2.

## Introduction

Almost half of older people admitted to hospital for acute illnesses, injuries or surgery will experience hospital-associated complications of delirium, functional decline, new incontinence, falls or pressure injuries [1]. These complications are more common in frailer people, often co-exist, and contribute to longer hospital stays, greater care needs, and higher mortality [1-4]. Consistent application of key age-friendly care principles including early regular mobility, adequate nutrition and hydration, and meaningful cognitive and social activities reduces delirium and may reduce other complications [5-7]. However, care practices supporting these key principles require cooperation and negotiation between healthcare staff from different disciplines and changes in practices and policies at individual, team and system level in response to multiple and varying local and organisational barriers [8-10]. Implementing age-friendly principles is thus a complex healthcare intervention [11, 12]. Design and reporting of complex interventions benefit from use of programme theory to explain core components and mechanisms (how the intervention is hypothesised to work, often articulated in a logic model), and implementation theory to describe the relationship between the intervention and the setting(s) (how it is implemented) [12-14].

Eat Walk Engage is a ward-based programme developed to improve multidisciplinary team delivery of age-friendly care principles (adequate nutrition and hydration, early regular mobility, and meaningful cognitive and social activities) to reduce hospital-associated complications in older inpatients [15]. It was developed, piloted and refined in a medical [16] and surgical ward [17] in a metropolitan teaching hospital. The logic model (Table 1) describes the hypothesised relationship between core components, activities, goals related to the principles, and clinical outcomes. Because facilitation was identified as a central component of pilot success [16], refinement and implementation of the program has been underpinned by the integrated Promoting Action on Research Implementation in Health Services (i-PARIHS) framework [18], which proposes that *facilitation* is the central activating mechanism for getting *innovation* (in this case, age-friendly care principles) into practice within the *context* (acute care wards) by engaging with the *recipients* (older patients and the multidisciplinary staff caring for them). The core components of Eat Walk Engage are a trained novice site facilitator, a local multidisciplinary work group, structured measures including older patient interviews and structured observations of care practices related to programme goals, and a trained multi-professional assistant. Informed by the i-PARIHS Facilitation Guide and facilitator’s toolkit [19] and supported by an experienced external facilitator, the site facilitator assesses the local context, measures current practice, and helps the work group to collaborate to initiate iterative cycles of improvement targeting the programme goals, including delegation of appropriate tasks to the multi-professional assistant [15]. Improvements may include changes at individual patient or provider level (e.g. ensuring a patient is wearing spectacles), team level (e.g. creating and using a cognitive kit of puzzles, games, craft resources and fiddle objects) or system level (e.g. procuring ward signage and calendar clocks to support orientation). Improvements are informed by older patients’ reported experience and suggestions, tailored to observed care practices and context and monitored by repeated measurement.

**Table 1:** Eat Walk Engage logic model (articulating the programme theory) with the corresponding measures reported in the implementation evaluation below, based on guidance for process evaluation of complex interventions in health care [13]. Patient outcomes are reported in the primary evaluation of the CHERISH cluster randomized trial [21]

Inputs (core components)	Intervention activities	Programme goals	Patient outcomes
• Eat Walk Engage site facilitator trained and mentored by expert facilitator • Ward-based multidisciplinary work group • Structured interviews and care process measures • Trained Eat Walk Engage assistant	Under guidance of the site facilitator, the work group: • identifies local barriers and enablers • develops shared improvement goals pertaining to the key principles • clarifies team roles and identifies opportunities for improvement • initiates small cycle improvements with re-evaluation	Improved team prioritisation Higher proportion of older patients achieve: • Early and adequate nutrition and hydration • Early mobility and independence • Meaningful cognitive and social engagement	Reduced hospital-associated complications leading to: • Reduced length of stay • Reduced facility discharge • Reduced 6-month readmission and mortality
Implementation	Context and implementation	Mechanisms of impact	
• Staff recruitment and training • Work group meeting frequency and attendance • Completion of interviews and audits	• Context mapping • Implementation strategies • Improvements	• Patient interviews • Behavioural mapping • Mealtime audits	

The Collaborative for Hospitalized Elders: Reducing the Impact of Stays in Hospital (CHERISH) trial [20] was a hybrid implementation-effectiveness trial evaluating Eat Walk Engage in four Queensland hospitals from January 2016 to May 2017. Effectiveness was evaluated using a cluster randomised trial design between October 2016 and April 2017, and demonstrated a significant reduction in hospital-associated delirium [21], consistent with other trials of multicomponent non-pharmacological interventions [5]. There were no significant changes in functional decline, incontinence, falls or pressure injuries, and there were promising but inconclusive findings for clinical outcomes (length of stay, facility discharge, and 6 month death and readmission) [21].

The aim of this implementation evaluation informed by the UK Medical Research Council guidance for evaluating complex interventions in healthcare [12, 13] and our logic model (Table 1) was to understand how Eat Walk Engage worked by

- describing context, implementation and improvements in each site;
- describing and analysing changes in process measures that are postulated mechanisms of impact leading to the observed patient outcomes;
- describing how facilitation supported tailored implementation as hypothesised by the underpinning i-PARIHS implementation framework; and
- proposing how key contextual features of different sites may have contributed to implementation success or failure [20]. This will help to build a more advanced understanding of how and why the programme works to inform spread and scale-up.

## Methods

### Setting

This study was initiated by hospital-based clinician researchers through a collaborative partnership with the Queensland Government, Queensland University of Technology, geriatric and health services research academics, and two Hospital and Health Services (HHS) in Queensland, Australia. The Queensland government funds universal free hospital care through public hospitals organised into 16 HHS. Senior executive leaders from one metropolitan and two regional HHS were invited as industry funding partners. One metropolitan and one regional HHS supported participation of four hospitals (two inner metropolitan, one outer metropolitan, one regional). Each hospital nominated two acute care wards where more than half of inpatients were aged over 65 years, the program was aligned with hospital priorities, and there was nurse unit manager agreement. One ward at each hospital was randomised to implement Eat Walk Engage, providing four intervention wards. Implementation commenced in January 2016.

### Participants

Participants for the implementation evaluation included inpatients on the four intervention wards before (October -December 2015) and after (March–May 2017) implementation, and multidisciplinary work group members and facilitators.

### Implementing Eat Walk Engage

Implementation drew on facilitation as experiential learning, led by two experienced facilitators who recruited, trained and mentored novice site facilitators using concepts and tools outlined in the i-PARIHS Facilitation Guide [19, 22]. The experienced facilitators were senior clinician researchers who had designed, implemented and evaluated the pilot Eat Walk Engage programme. They were supported by experts in implementation science and practice not involved in delivery of the programme, including an expert consumer advocate. They worked with local clinical leaders at each site to recruit site facilitators, who were mid-career nurses or allied health professionals selected through a competitive recruitment process focussing on skills and attributes of facilitation (e.g. self-awareness, communication, interpersonal and assessment skills) [19, 23, 24]. The experienced facilitators provided four half-day initial group training sessions for site facilitators (October to December 2015) including didactic and interactive content based on the i-PARIHS Facilitation Guide [19], evidence for age-friendly care principles and the prevention and management of hospital-associated complications, and provision of key readings. Mentoring included monthly half-day face-to-face peer group meetings (January 2016 to May 2017), with telephone and email support available between meetings, supporting debriefing, reflection on practice and shared learning [25]. The experienced facilitators visited each site 3–6 times before and during implementation, meeting key stakeholders and participating in local work group meetings. Project funding supported 24 h per week experienced facilitator time to support project management and external facilitation across all sites, and 16 h per week site facilitator plus 20 h per week multi-professional assistant for each implementation ward.

Site facilitators engaged with the nurse unit manager and key staff on each intervention ward to form a work group focussed on improving care of older people. The work group included champions, opinion leaders and/or key roles for improving care of older people (e.g. nursing staff, nurse educator or clinical facilitator, physiotherapist, dietitian, occupational therapist, medical staff, patient support staff). Site facilitators scheduled monthly meetings and helped work group members reflect on evidence, tacit knowledge, older patient interviews and structured observations of care practices (see below). They supported the work group to identify and prioritise areas for improvement, clarify roles and relationships, and trial cycles of improvement aligned with programme goals, agreed team priorities and available resources.

Multi-professional assistants were allied health or nursing assistants selected by interview who received two weeks of training in care of older people, including a work instruction manual, face-to-face training with local allied health professionals (e.g. physiotherapist, speech pathologist) and work shadowing with an experienced assistant. Any clinician could delegate tasks aligned with programme goals (e.g. setting patients up for a meal, supporting supervised mobility or exercise activities, providing assisted listening devices). Assistants could also assist with ward-level tasks related to environment and resources (e.g. updating orientation boards).

### Evaluating the implementation

The implementation evaluation was a prospective, multi-site evaluation using a theory-based research perspective to describe the context, implementation and mechanisms at each site [12]. The evaluation was guided by the UK Medical Research Council guidance for process evaluation of complex interventions [13]. The intervention and causal assumptions were articulated in our logic model (Table 1). The i-PARIHS framework [18] informed implementation (with a central emphasis on facilitation) and context assessment. Pre-intervention measures were collected by the site facilitators in October-December 2015 before implementation commenced and presented to the local work group to prioritise and inform improvements. Post-intervention measures were repeated in March–May 2017 by trained staff not involved in implementation on that ward. Pre-intervention context and process measures (patient interviews and structured observations of care practices, described in detail below) informed and guided local implementation and improvements, and also provided a baseline for comparison with the post-intervention process measures.

*Context* was assessed by each site facilitator using a spreadsheet tool based on the i-PARIHS facilitation checklist [19], focussing on determinants of implementation at the level of intervention, recipients and inner (ward) context. Each site facilitator synthesised their observations from patient interviews and structured observations (described below), group and individual discussions with local staff members and informal observations of ward practices and culture, to identify and rate barriers and enablers, supported by reflective discussions with the experienced facilitators and peer site facilitators. Contextual determinants were scored from -2 (major barrier) to + 2 (major enabler) with brief explanatory comments [22]. Scores and comments were mapped and reviewed pre-implementation, mid-implementation and after implementation, and compiled into a summary table by the experienced facilitators.

*Implementation* described adherence to the core intervention components (including staff recruitment and training, work group attendance and completion of interviews and structured observations of care practices); the *implementation strategies* used by facilitators to enable change; and the resulting individual, team and system-level *improvements* addressing programme goals. Data were obtained from minutes and field notes maintained by the experienced facilitators during regular group face-to-face meetings with the site facilitators and electronic minutes maintained by site facilitators during their local work group meetings. The two experienced facilitators independently extracted and analysed information using directed content analysis [26], immersing in the textual data and applying codes derived from theory and the pre-defined program logic. Implementation strategies used by facilitators were coded deductively using the Expert Recommendations for Implementing Change (ERIC) taxonomy [27]. Improvements were coded based on the programme goals (‘Eat’, ‘Walk’, ‘Engage’ and team communication, Table 1) and summarised for each ward, including the improvement, team member(s) responsible, the barrier or enabler being addressed and the level of intervention (individual, team or system). The consensus summaries were discussed within the broader research team which included two site facilitators and four external researchers.

*Mechanisms of impact* were evaluated by process measures before and after the intervention was implemented, including older patient interviews and structured observations of care practices aligned with the programme logic model (Table 1). Site facilitators received group and individual training from the experienced facilitators including supervised practice of all care practice measures. Semi-structured patient interviews were conducted with a purposeful sample of inpatients likely to benefit from this ward-based intervention [20], aiming to sample approximately ten consenting inpatients aged 65 years or more with a length of stay of 3 days or more on each ward, excluding patients with critical illness, severe cognitive impairment or at end of life who have unique considerations in their care. Questions included perceived importance of each key principle (mobility, nutrition and meaningful engagement) during acute hospitalisation, using a 4-item response scale (very important, somewhat important, not really important, important but unable to do) and whether participants had received recommendations from their health care team about each principle, reflecting team communication. Responses to open-ended questions about barriers, enablers and suggested improvements were summarised for feedback to the work group at each site and have been reported in detail elsewhere [8].

Cross-sectional structured mealtime observations were undertaken for all ward inpatients (excluding patients who were fasting, receiving enteral or parenteral nutrition, off the ward at mealtimes or receiving end of life care) at three mealtimes (breakfast, lunch and dinner) during a 1–2 week period. The observer noted whether each patient was sitting up when the meal arrived, had the tray table in reach, and was interrupted during the meal. They recorded whether patients required assistance with meal set-up or eating, and whether they received this assistance within 10 minutes. We evaluated the percentage of mealtimes where patients were sitting in a chair and received timely assistance, as these factors are associated with increased nutritional intake [28].

Structured observations of patients’ physical, cognitive and social activities were conducted using activity mapping [29, 30], systematically sampling patient activities on one weekday on each ward. All patients in a room (except those receiving end of life care) were observed for two minutes before moving to the next room in continuous sequential observations between 8am and 4 pm, sampling each patient every 20–30 min depending on ward size and room configuration. The highest level of activity during each observation period was recorded using a hierarchical tool encompassing location, physical activity, cognitive/social activity, and company. We averaged the percentage of observations at each level for patients with at least 4 h of observation (excluding patients who were discharged early, admitted late or off the ward for more than half the day) [29, 30]. We evaluated the percentage of observations where the patient was standing or walking and the percentage where they were engaged in cognitive or social activities (e.g. talking to others, reading, watching television) aligned with programme goals. Increased physical and cognitive activities may reduce delirium and functional decline in hospital [5, 7].

### Analysis

Context measures were summarised for each ward and over time in a table to allow visual comparison. Implementation strategies were summarised in a table using the ERIC taxonomy [27] and describing the level of facilitator responsible (experienced vs novice) for implementing each strategy, while improvements were summarised by site, focussing on the persons responsible for each strategy and the barrier being addressed to illustrate how approaches were tailored to local context. Care practice measures capturing the program goals (Table 1) were summarised descriptively pre- and post-implementation at ward level in tables and graphs to illustrate site-level changes, and averages across implementation wards were compared before and after implementing the intervention to test program-level process improvement changes and understand their potential impact on program-level outcomes as reported in the effectiveness evaluation. The researcher team triangulated contextual information, improvement strategy ‘dose’ and observed care practice changes within the case studies of each ward through drafting and discussion of findings to develop consensus propositions about the variable implementation success across sites. Measures to enhance trustworthiness of analysis included use of established well-defined coding frameworks for context assessment and implementation strategies and dual coding of implementation and improvement strategies (dependability); data collection triangulation, researcher triangulation and regular peer debriefing regarding methods and findings with a team which included members internal and external to the implementation team (credibility); and thick description of context (transferability) [31].

## Results

### Context

**Fig. 1:**
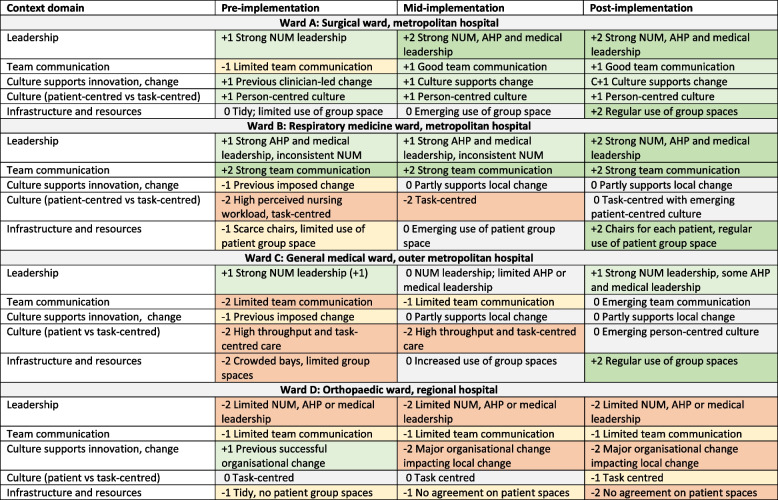
Summary of selected features of the ward context on each ward identified by the Eat Walk Engage facilitators at baseline, mid-implementation and end of implementation. Green cells represent enablers while orange cells represent barriers. Numbers indicate strength of barrier (negative) or enablers (positive). AHP Allied Health Professional; NUM Nurse Unit Manager

The four intervention wards were a general surgical, respiratory medicine, general medicine, and orthopaedic surgery ward. Each ward had 26–38 beds in combinations of multi-bed bays and single rooms. Patients were cared for by junior medical officers supervised by consultant physicians or surgeons, registered and enrolled nurses with daytime ratios of 4–6 patients per nurse, and allied health professionals (physiotherapists, occupational therapists, dieticians, social workers, etc.) with varying team communication structures. Figure 1 summarises selected context domains before, during and after implementation, illustrating contextual barriers between wards and over time. Initially Site A had strong nursing leadership, person-centred culture and some experience with clinician-led improvement but did not have strong interdisciplinary communication. Site B had strong interdisciplinary communication but inconsistent nursing leadership and a task-centred nursing culture, with little improvement experience. Site C had strong nurse unit manager leadership but a high workload and task-centred culture, with little improvement experience and limited interdisciplinary communication, while Site D had some successful organisational change experience but limited nursing leadership and interdisciplinary communication, and a task-centred culture. All sites had a lack of group spaces used for patients. Over time, sites A, B and C showed fewer contextual barriers, but Site D showed greater barriers, likely related to powerful competing priorities of an impending move to a new campus.

### Implementation

Site facilitators were an occupational therapist, two dietitians and a registered nurse, all with four or more years of clinical experience and previous quality improvement experience. Each was working within the site in a clinical capacity which provided some tacit knowledge of the local and organisational context. One had previous experience working in Eat Walk Engage at the pilot site. All attended four half-day group training sessions, and 10–12 group mentoring sessions, and all remained in the position until the end of the evaluation. Multidisciplinary work groups were convened in all sites by February 2016. During the following 15 months, Site A held 11 work group meetings, Site B held 16, Site C held 9 and Site D held 5. Median attendance was 6–8 staff per meeting at all sites. Assistants were recruited at three sites by October 2016, and included a physiotherapy assistant, an occupational therapy assistant and a nursing assistant. All completed the introductory training and work shadowing; the nursing assistant required additional mobility training with the local physiotherapist. No assistant was recruited at site D.

Facilitators employed multiple implementation strategies to facilitate change [27] as outlined in detail in Table 2. Experienced facilitators initiated an implementation advisory group and maintained relationships with site leaders and key clinicians. They provided training and support to site facilitators, including data management and templates for data reporting and feedback. Site facilitators developed relationships with ward staff and identified local champions to inform, initiate, evaluate and sustain improvements. They involved older patients and carers through interviews, development of patient/carer information and consultation about improving the environment. They collected and fed back data to inform improvements, negotiated role delineation and responsibilities, facilitated improvements, and supported implementation of the assistant role. They provided opportunities for teams to celebrate progress.

**Table 2:** Implementation strategies (classified and defined based on the ERIC compilation) [27] used by the experienced and novice site facilitators

**Strategy group**	**Strategies**	**Comments**
Provide interactive assistance	Facilitation	The core strategy was enabling facilitation
Develop stakeholder inter-relationships	• Inform local opinion leaders • Obtain formal commitments • Develop academic partnerships • Identify and prepare champions • Recruit, designate and train for leadership • Use advisory group • Use an implementation advisor • Visit other sites • Promote network weaving • Organise clinician implementation team meetings • Capture and share local knowledge • Conduct local consensus discussions • Identify early adopters	**Experienced facilitators** identified key executive and clinical leaders in each site through existing networks and arranged contracts within an academic-industry partnership grant. They identified senior clinical managers to support the local program and assist recruiting program staff (site facilitators and assistants). They established an implementation steering committee, including an expert facilitator. They promoted site visits and shared events for program staff to nurture a shared identity. **Site facilitators** identified ward-level opinion leaders and champions to support changes. They formed a work group to share local knowledge, which was used along with information from patient interviews and care process measures to develop a shared vision and prioritise improvements. They supported team members to lead changes that aligned with the key principles. They developed relationships with experienced and peer novice facilitators and made site visits to other intervention wards.
Train and educate stakeholders	• Develop educational materials • Make training dynamic • Conduct ongoing training • Conduct educational outreach visits • Create a learning collaborative • Use train-the trainer strategies • Shadow other experts • Provide ongoing consultation • Conduct educational meetings	**Experienced facilitators** developed interactive education materials for site facilitators based on the i-PARIHS Facilitator’s Guide and key program principles. They provided monthly face-to-face training and support including role modelling facilitation of work group meetings at sites. They provided program overview information to executive and clinical leaders at each site and developed training resources for multi-professional assistant including work shadowing opportunities. They were available by telephone or email throughout the program. **Site facilitators** provided education to ward clinicians about the program’s key principles, and facilitated relevant education on topics identified by the work group
Use evaluative and iterative strategies	• Conduct local needs assessment • Assess for readiness, identify barriers and enablers • Obtain and use patient feedback • Audit and provide feedback • Conduct cyclical small tests of change • Purposely re-examine the implementation	**Experienced facilitators** trained the novice site facilitators to complete context assessments, interviews and care process measures, and helped them to reflect on these data to create a narrative to inform and inspire the work group. They provided support for managing, analysing and presenting data to the work group, and created site progress reports at the end of implementation which they fed back to senior clinicians and managers. **Site facilitators** used formal and informal staff discussions, patient interviews, audits and personal observations to complete a local context assessment. They provided feedback on interviews and care process measures to the work group to stimulate suggested improvement strategies, and reassessed periodically
Adapt and tailor to context	• Tailor strategies • Promote adaptability • Use data experts	**Experienced facilitators** assisted site facilitators to develop data reports and narratives **Site facilitators** supported teams to implement improvement strategies aligned with the key principles which were adapted to context.
Support clinicians	• Develop resource sharing agreements • Create new clinical teams • Remind clinicians • Revise professional roles	**Experienced facilitators** developed the contracts which supported co-funding of the additional roles **Site facilitators** used regular work group meetings to maintain awareness, and supported clinician reminder strategies for improvements (e.g. poster, in-service education). Some improvement strategies included clarification and redistribution of roles, and delegation to the assistant
Engage consumers	• Involve older patients • Prepare older patients and families to be active participants	**Experienced facilitators** engaged a consumer on the implementation steering committee. **Site facilitators** involved patients through structured interviews and encouraging other feedback mechanisms (e.g. suggestion box, patient/family brochures)
Change infrastructure	• Change physical structure and equipment	**Site facilitators** helped work groups to advocate for appropriate clinical resources identified as necessary to meet key principles (e.g. suitable chairs, patient lounge, cognitive materials)

Using these strategies, the site facilitators facilitated 45 discrete improvements summarised in Table 3. These included 10 changes to individual care practices (e.g. physiotherapist sitting patients out for lunch after their morning walk), 20 changes to team processes (e.g. allied health professionals providing additional nursing education about the key principles) and 15 changes to systems (e.g. changing the time of meal delivery to ensure nursing staff were available to assist). Improvements were mostly led and delivered by ward staff, with some delegated to the assistants. Some system-level improvements (e.g. engaging volunteers) required direct actions by the site facilitator. Site A implemented 28 improvements, sites B and C each implemented 23 and site D implemented nine. Fifteen improvements were implemented across 3 or more sites, including six related to nutrition, five to cognitive and social engagement, two to mobility and two to team communication.

**Table 3:** Improvement strategies undertaken at each site to address programme goals

Programme goal	Intervention and team member(s)	Barrier/enabler being addressed	Intervention level	Ward A	Ward B	Ward C	Ward D
Eat	MDT organise group morning tea	Activities	Team	✓		✓	
Eat	MPA provides mealtime assistance	Assistance	Individual	✓	✓	✓	
Eat	Physio/OT assist sitting patients out for meals	Assistance	Individual	✓	✓	✓	
Eat	Facilitator negotiates change to time of meal delivery	Competing priorities	System		✓		✓
Eat	Admin assistant rings bell to notify staff when meals arrive	Competing priorities	System			✓	
Eat	Nurses add prompts to nurse planning documents	Competing priorities	System		✓	✓	
Eat	Nurses revise workflow at meal time	Competing priorities	Team			✓	
Eat	Nurse unit manager changes AIN shift time to support meals	Competing priorities	Team		✓		
Eat	Nurse unit manager changes timing of nursing breaks	Competing priorities	Team			✓	
Eat	MDT advertise mealtimes to staff	Competing priorities	Team	✓	✓	✓	
Eat	Nurse unit manager reprioritises AIN tasks	Competing priorities	Team	✓	✓	✓	
Eat	Facilitator negotiates broader indications for HPHE meals	Food availability	System			✓	
Eat	Dietitians negotiate availability shelf stable meals	Food availability	System	✓			
Eat	MDT advertise mealtimes to patients and families	Including patients and families	Team	✓	✓	✓	
Eat	MDT encourage family involvement	Including patients and families	Team	✓		✓	
Eat	Dietitians provide staff in-service education	Knowledge/skills	Team	✓			
Eat	Senior nurse leads rounds to sit patients out for meals	Leadership	Team		✓		
Eat	Nurse unit manager reminds staff about prioritising meal times	Leadership	Team	✓	✓	✓	✓
Walk	MPA provides exercise group	Activities	Individual			✓	
Walk	MPA mobilises patients	Assistance	Individual	✓	✓	✓	
Walk	AIN mobilises patients	Assistance	Individual	✓			
Walk	Facilitator improves patient lounge	Destination	System	✓	✓	✓	
Walk	MDT provide map and/or markers for way finding	Destination	System		✓		✓
Walk	MDT sets up additional walking destination	Destination	System	✓		✓	
Walk	Facilitator negotiates chair purchases	Equipment	System		✓		
Walk	Facilitator arranges clothing donations	Equipment	System	✓			
Walk	Nurses inform patients and families re patient lounge	Including patients and families	Individual		✓		
Walk	Physios provide nursing in-service on safe mobilisation	Knowledge/skills	Team			✓	
Engage	MPA supports activities	Activities	Individual	✓	✓	✓	
Engage	MDT or nurses lead activity groups	Activities	Team	✓	✓	✓	
Engage	Facilitator engages volunteers as patient companion or for groups	Activities	System	✓	✓	✓	
Engage	Facilitator negotiates discounted TV access	Activities	System	✓			
Engage	Facilitator negotiates patient library	Equipment	System		✓		
Engage	Facilitator sources sensory aids e.g. glasses	Equipment	System				✓
Engage	Team sources daily newspaper	Equipment	Team		✓		✓
Engage	MDT purchase or donate cognitive resources e.g. puzzles, games, pencils	Equipment	Team	✓	✓	✓	✓
Engage	Nurse unit manager provides cognitive resource trolley or cupboard	Equipment	Team	✓	✓	✓	✓
Engage	Nurses provide activity table	Equipment	Team	✓			
Engage	MDT use tools for cognitive impairment e.g. biography tool, orientation boards	Including patients and families	Individual	✓		✓	
Engage	Nursing director changes visiting hours	Leadership	System	✓			✓
Team communication	MDT provides patient brochures	Including patients and families	Individual				
Team communication	MDT use patient goal board	Including patients and families	Individual	✓	✓		
Team communication	Nurse unit manager creates staff suggestion box	Leadership	Team	✓	✓		✓
Team communication	MDT increase meeting frequency	Team communication	Team	✓			
Team communication	MDT create delegation methods to MPA	Competing priorities	Team	✓	✓	✓	
Total interventions				28	25	25	9

*MDT* Multidisciplinary team, *MPA* Multi-professional assistant, *OT* Occupational therapist, *AIN* Nursing assistant, *HPHE* High protein high energy, *TV* Television

### Mechanisms of impact

**Fig. 2:**
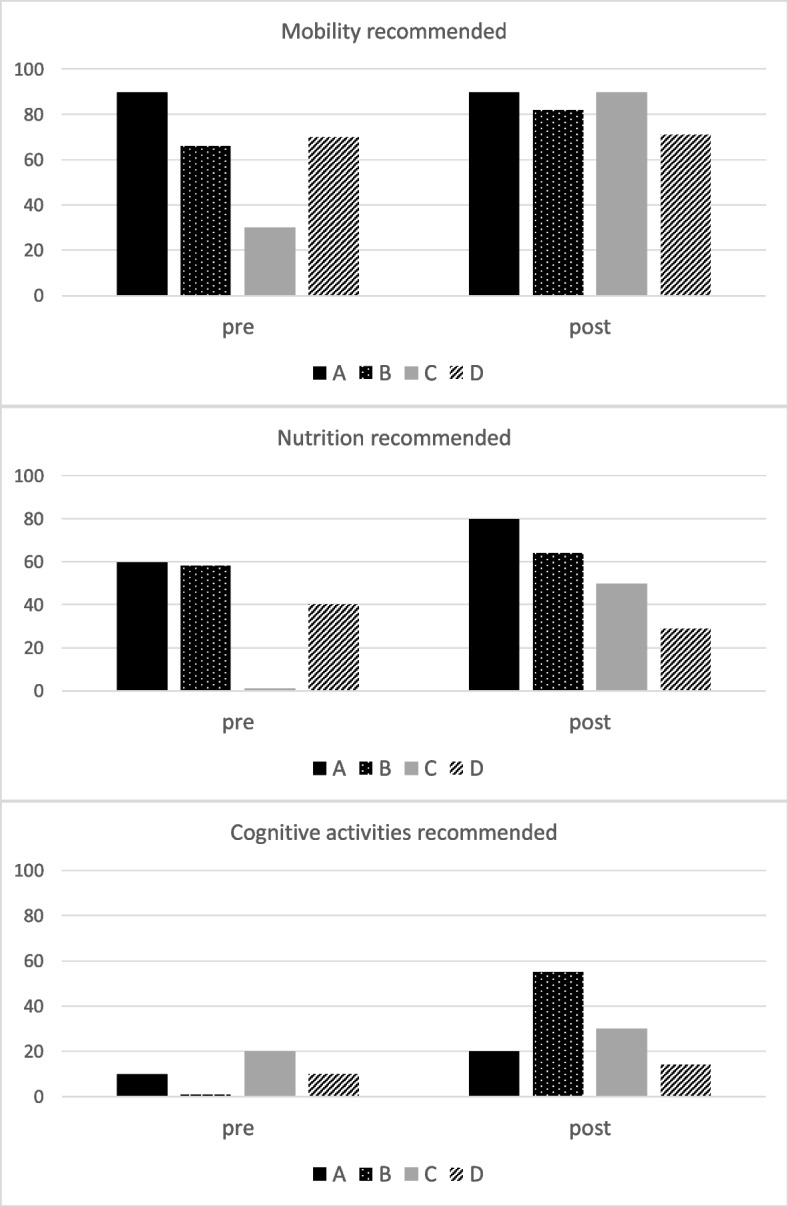
Percentage of older patient interviewees (pre *n* = 42; post *n* = 38) who recalled their doctor or other member of the health care team providing recommendations related to mobility, nutrition and cognition

Interviews were conducted with 42 older inpatients pre-intervention and 38 post-intervention (Site D only achieved 7 post-intervention interviews). Most agreed that the key principles were very important to recovery (Additional file 1). Figure 2 illustrates the percentage of interviewees who received recommendations related to the key principles by site. More reported receiving recommendations about mobility than nutrition or cognition. Following implementation, participants were more likely to have received recommendations from their healthcare team in sites A, B and C, with no change in Site D.

**Fig. 3:**
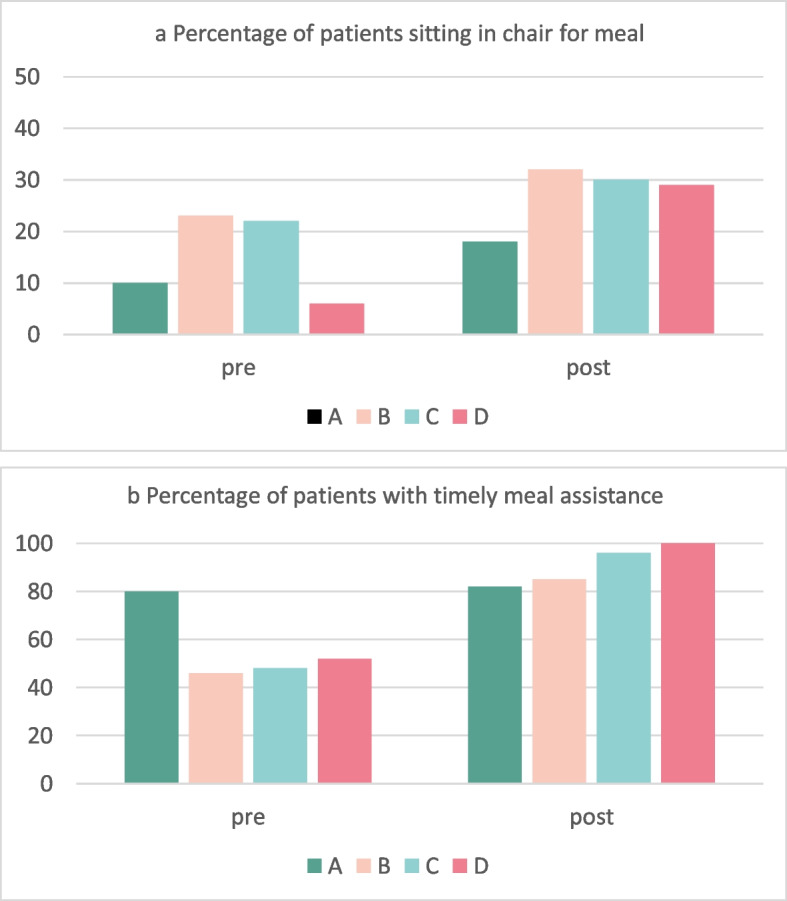
Structured observations of mealtime care practices before and after implementing Eat Walk Engage, by site. The percentage of patients sitting out when the meal arrived (3a) and receiving mealtime assistance if required (3b) was obtained from non-participant observation of three mealtimes (breakfast, lunch and dinner; pre *n* = 283; post *n* = 297)

Mealtime observations pre- and post-intervention are summarised in Fig. 3a and b and Additional file 1. There was substantial variation between sites initially. Patients sitting in a chair when the meal arrived increased from 47/283 (17%) to 83/297 (28%). Overall, 94/283 (33%) of inpatients pre-intervention and 63/297 (21%) post-intervention required set-up or eating assistance. Timely assistance increased from 53/94 (56%) pre-intervention to 58/63 (92%) post-intervention. All sites showed some improvements.

**Fig. 4:**
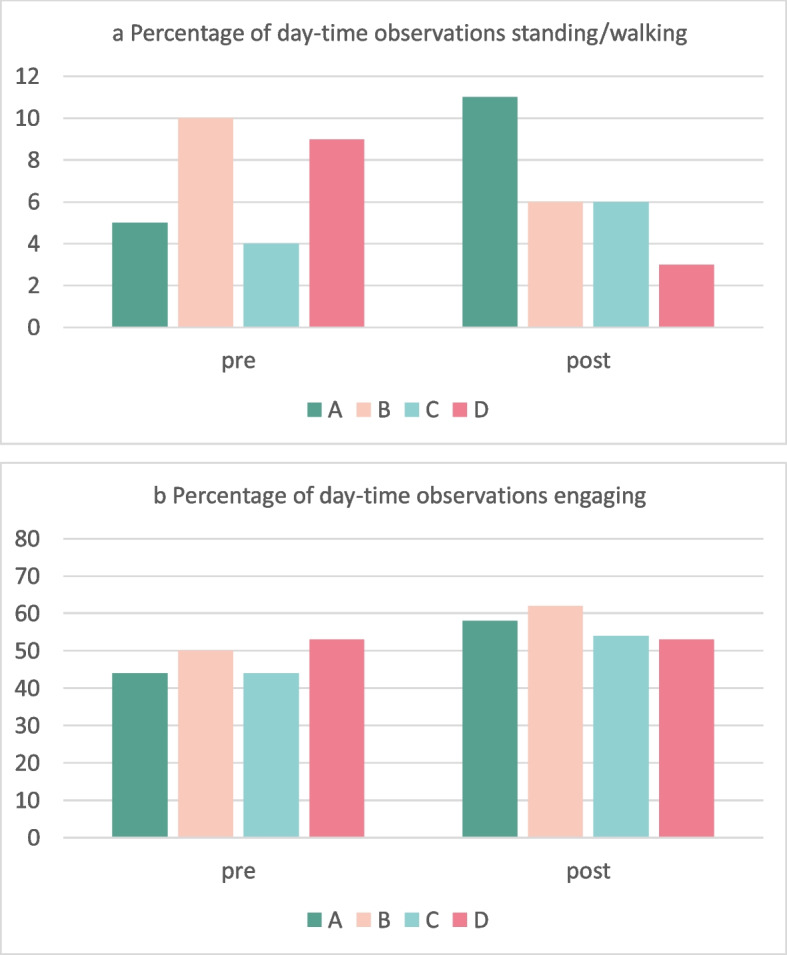
Structured observations of physical and cognitive activities before and after implementing Eat Walk Engage, by site. The average percentage of patient observations standing or walking (4a) and the average percentage of patient observations spent engaging in physical or cognitive activities (4b) obtained from 8 h of continuous daytime observation using behavioural mapping (pre *n* = 85; post *n* = 111)

Figure 4a and b summarise observations from activity mapping. The mean proportion of patient time walking or standing did not change overall (101/1431 [7%] observations of 85 patients pre-intervention and 152/2202 [7%] observations of 111 patients post-intervention). Sites A and C recorded greater mobility post-intervention, but Site B and Site D showed less. The percentage of time spent engaged in social or cognitive activities increased from 712/1496 (48%) observations of 85 patients pre-intervention to 1308/2293 (57%) observations of 111 patients post-intervention. Increases were seen in Sites A, B and C.

## Discussion

This paper describes implementation of Eat Walk Engage on medical and surgical wards in four hospitals. The process evaluation illustrates the complexity of characterising, implementing, and evaluating flexible multi-component health care interventions in varying contexts [12]. Core components were clearly specified but implementation strategies and improvements were adapted through active facilitation, informed iteratively by older persons’ perceptions and suggestions, observations of existing care practices, and dynamic features of each site’s context, in keeping with emergence properties of complex systems, which can be difficult to capture in programme theory [32]. The multiple levels of leadership and agency illustrated in this evaluation (including experienced and novice site facilitators, work group members, assistants, other staff and families) provide an example of how differentiating implementation and improvement strategies, or implementers and participants, may be somewhat artificial in complex systems [13]. There was substantial variation in number and type of improvements between sites, and the site with lowest fidelity to the core components had fewer improvements and less care practice changes, a ‘dose effect’ which supports our logic model.

The evaluation demonstrated improvements in many care practices within 15 months of implementation. Older patients recalled more recommendations about the key principles, suggesting improved team communication. Structured observations of care practices demonstrated improvements in mealtime care and participation in social and cognitive activities, but not consistent improvements in mobility. Mobility-related improvement strategies were observed less consistently across sites than nutrition and engagement strategies, which might help explain this difference. The facilitators’ backgrounds (dietetics, occupational therapy and nursing) may have meant that they were more confident or comfortable supporting nutrition or engagement interventions, or the well-recognised multi-level barriers to in-hospital mobility [10, 33] may have made changes in this domain more difficult to achieve. The observed process improvements are plausible mediators of the significant reduction in delirium demonstrated in the effectiveness trial [21]. A recent systematic review of non-pharmacological interventions for delirium prevention highlighted the importance of re-orientation, cognitive stimulation, and attention to nutrition and hydration [5]. In contrast, inconsistent mobility improvements could explain why hospital-associated disability and incontinence were not significantly reduced [34, 35]. A longer intervention period or earlier prioritisation of mobility improvement strategies might increase the impact of this program on these outcomes.

The study supports the i-PARIHS implementation theory, illustrating how experienced facilitators supported novices to consciously adapt implementation strategies and improvements within varied and dynamic contexts. Facilitation is both a role and a set of activities [36]. The study funded a dedicated site facilitator within each ward, whose knowledge and skills were supported by training, mentoring, and opportunities for peer reflection [19, 22, 37]. Detailed description of implementation strategies and improvements makes the facilitation process visible [38], illustrating the diverse activities required of a facilitator [36, 37]. This case study of experiential learning adds to facilitation theory [39] by describing distinct roles of the experienced and novice facilitators. The experienced facilitators worked as an external-internal facilitator and boundary spanner to build organisational capacity [38, 40] while the site facilitator worked as a clinical practice facilitator to support the clinical teams and assistant to trial specific improvements at individual and team level. A previous theory-based implementation evaluation of a delirium prevention program highlighted the importance of a dedicated facilitator to support iterative practice change in a dynamic environment [41]. However our analysis also describes the important role of the experienced facilitator, who not only trained and supported site facilitators [23, 25], but also engaged in their own facilitation roles including understanding and engaging key stakeholders within the organisational and outer context (beyond the influence of the novice facilitator), actively supporting data collection and reporting, and enabling networking between sites and stakeholders. Our findings suggest these roles need to be recognised and resourced for successful scale and spread of this complex intervention [41].

Our longitudinal observations also illustrate how context influenced and was influenced by implementation [42]. An adverse and deteriorating context at site D was associated with challenges to adoption of the core components (i.e. fidelity), fewer team-led improvements and limited change in care practices. Site executive leaders had committed to the trial before relocating to a newly built facility, which occurred in early 2017 as the trial was finishing. However, this major competing priority created impacts on staff recruitment, leadership and staff morale which could not be mitigated by the facilitators or local staff, and hampered success, similar to experiences reported by other investigators [41]. Sites B and C had adverse initial features in their inner organisational context, but team communication, culture and infrastructure became more positive as site facilitators built trust and teamwork, encouraged reflection on care practices, and supported iterative improvements. Combining local tacit knowledge with shared reflection on practice to create tension for change empowers multi-level, distributed leadership which may be more effective than traditional hierarchical leadership within complex healthcare systems [43, 44]. Sustaining this complex program will require continuing skilled facilitation and regular reassessment of context, to allow dynamic adaptation to changes in personnel, resources, leadership and organisational priorities [45]. Although our research design was pragmatic, implementing and sustaining the program outside of a research agreement may raise new challenges in engaging and sustaining organisational leadership and visibility.

Strengths of this study are a clear logic model and prospective use of an appropriate implementation theory. Implementation was guided by older patients’ experience and local context as well as published evidence, and ward-level observations provided meaningful measurement of programme goals both as an opportunity for improvement and a measure of progress. Rich evaluation using multiple data sources captured critical concepts of context, implementation and mechanisms; an accompanying in-depth qualitative evaluation of the facilitator’s journey will augment the evaluation further. We acknowledge potential limitations, including the challenges inherent in program developers and facilitators being involved as investigators [13]. The evaluation group included the two experienced facilitators and two novice site facilitators, but also included senior investigators not involved in the implementation and a consumer representative, and collaborative implementation group meetings for review of analysis methods and emerging findings supported researcher triangulation to enhance credibility as well as interpersonal, methodological and contextual reflexivity to reduce potential bias [46]. The facilitator-investigator role provides valuable understanding of the breadth of implementation strategies and improvements, which could otherwise remain invisible and lead to challenges with sustainability [47]. Our context assessments focussed most closely on the inner organisational level because the ward is the level of the intervention and site facilitator influence; while we recognised the major impact of specific outer organisational factors in Site D, there may have been other under-recognised outer organisational factors such as leadership, culture and cosmopolitanism that influenced implementation in other sites. We did not formally test inter-rater reliability for the process measures, and there is not yet sufficient published data to establish meaningful change in these measures. Implementation strategies and improvements were obtained from content analysis of project documents and field notes, which may have led to incomplete recording of strategies and may reduce dependability. This could be mitigated in future studies by designing tools for prospective, real time tracking of how implementation strategies and improvements are tailored, which has been recognised as a missing enabler for operationalising i-PARIHS and other implementation frameworks in practice [22].

## Conclusions

The Eat Walk Engage programme was implemented with reasonable success across four wards with varied and dynamic contexts using an experienced-novice facilitation model. The facilitators used a wide range of implementation strategies to support diverse improvements aligned with program goals, and these were associated with improvements in several key processes of age-friendly care which likely mediated the observed reduction in delirium. The limited impact on functional decline and length of stay might be explained by inconsistent improvements in mobility, perhaps because insufficient improvements were implemented within the project time frame to address this care practice. Spread and sustainability of this complex intervention will require continued investment in skilled multi-level facilitation to support clinician-led improvement in dynamic and varied ward contexts.

## Supplementary Information

**Additional file 1: Supplementary Table 1.** Summary of patient interview data regarding perceived importance of the key principles, and their recollections of staff recommendations related to these principles. **Supplementary Table 2.** Summary of key process of care measures before and after implementation, by site.

## Data Availability

The datasets generated and analysed in this research are not publicly available in accordance with local ethics approval, but are available from the corresponding author on reasonable request.

## Glossary

**Context**: factors external to the intervention that may influence its implementation e.g. culture, resources, leadership, learning environment [13, 18]
**Core components**: active ingredients of the intervention which are hypothesised (or proven) to be essential for intervention success
**Facilitation**: making things easier; may encompass a wide range of implementation strategies [26]. It is the construct that activates implementation, through assessing and responding to characteristics of the intervention (innovation), the individuals involved (recipients) and the context [18].
**Facilitator**: a designated role with attributes, skills, knowledge and support to enact facilitation. May be internal or external to the setting in which the evidence is being implemented, and may range in experience from novice to expert [18].
**Intervention**: the evidence-based practice or program being implemented, in this case the Eat Walk Engage program
**Improvements**: changes to individual or shared activities or resources intended to increase the likelihood of achieving the program goals
**Implementation**: systematic uptake and integration of evidence-based practice or programs into practice to improve healthcare quality and effectiveness [14].
**Implementation framework**: a theoretical approach that aims to understand and/or explain what influences implementation outcomes [14]. In this case we used the i-PARIHS (Integrated Promoting Action on Research Implementation in Health Services) implementation framework [18] which has been widely used to plan, guide and/or evaluate how complex evidence is integrated into multi-disciplinary practice
**Implementation strategies**: Methods or techniques to enhance implementation of evidence-based interventions, in this case described using the ERIC (Expert Recommendations for Implementing Change) taxonomy designed to improve design and reporting of implementation strategies [26].
**Mechanisms of impact**: the way in which the improvements in individual or shared activities or resources are hypothesised to lead to improved clinical outcomes [13], in this case increases in nutrition, mobility and meaningful engagement.
**Tailoring**: adapting implementation strategies and/or improvements to address barriers and leverage enablers identified in the local context [26].
