# Supplementary material for: Implementing a ward-based programme to improve care for older inpatients: process evaluation of the cluster randomised CHERISH trial
Source: BMC Health Serv Res. 2023 Jun 21;23:668. doi: 10.1186/s12913-023-09659-2 (PMC10283300; doi:10.1186/s12913-023-09659-2)
Supplement: Supplementary file 1 — Additional file 1: Supplementary Table 1. Summary of patient interview data regarding perceived importance of the key principles, and their recollections of staff recommendations related to these principles. Supplementary Table 2. Summary of key process of care measures before and after implementation, by site. [file 12913_2023_9659_MOESM1_ESM.docx]

Additional File 1 “Implementing a ward-based programme to improve care for older inpatients: process evaluation of the cluster randomised CHERISH trial”

Supplementary Table 1: Summary of patient interview data regarding perceived importance of the key principles, and their recollections of staff recommendations related to these principles

|  | Rated ‘very important’ by patient, n (%) | | | Recall recommendation by healthcare team, n (%) | | |
| --- | --- | --- | --- | --- | --- | --- |
|  | Mobility | Nutrition | Cognition | Mobility | Nutrition | Cognition |
| *Pre-implementation* | | | | | | |
| Site A (n=10) | 10 (100) | 7 (70) | 8 (80) | 9 (90) | 6 (60) | 1 (10) |
| Site B (n=12) | 7 (58) | 10 (83) | 8 (66) | 8 (66) | 7 (58) | 0 (0) |
| Site C (n=10) | 6 (60) | 7 (70) | 9 (90) | 3 (30) | 0 (0) | 2 (20) |
| Site D (n=10) | 8 (80) | 10 (100) | 10 (100) | 7 (70) | 4 (40) | 1 (10) |
| ***All sites (n=42)*** | ***31 (74)*** | ***34 (81)*** | ***35 (83)*** | ***27 (64)*** | ***17 (40)*** | ***4 (10)*** |
| *Post-implementation* | | | | | | |
| Site A (n=10) | 10 (100) | 9 (90) | 10 (100) | 9 (90) | 8 (80) | 2 (20) |
| Site B (n=11) | 9 (82) | 10 (91) | 9 (82) | 9 (82) | 7 (64) | 6 (55) |
| Site C (n=10) | 8 (80) | 9 (90) | 8 (80) | 9 (90) | 5 (50) | 3 (30) |
| Site D (n=7) | 6 (86) | 5 (71) | 5 (71) | 5 (71) | 2 (29) | 1 (14) |
| ***All sites (n=38)*** | ***33 (87)*** | ***33 (87)*** | ***32 (84)*** | ***32 (84)*** | ***22 (58)*** | ***12 (32)*** |

Supplementary Table 2: Summary of key process of care measures before and after implementation, by site

|  | Meals where patient was sitting in chair when meal arrived, n (%) | Meals where patient was assisted within 10 minutes if required, n (%) | Daytime observations where patient was standing, walking or actively wheeling, n (%) | Daytime observations where patient was engaged in social or cognitive activities, n (%) |
| --- | --- | --- | --- | --- |
| ***Pre-implementation*** | | | | |
| Site A | 6/63 (10) | 12/15 (80) | 24/548 (5) | 222/503 (44) |
| Site B | 22/96 (23) | 14/30 (47) | 35/345 (10) | 171/344 (50) |
| Site C | 16/72 (22) | 14/24 (58) | 12/286 (4) | 127/289 (44) |
| Site D | 3/52 (6) | 13/25 (52) | 30/342 (9) | 192/360 (53) |
| ***All sites*** | ***47/283 (17)*** | ***53/94 (56)*** | ***101/1431 (7)*** | ***712/1496 [48]*** |
| ***Post-implementation*** | | | | |
| Site A | 11/62 (18) | 9/11 (82) | 54/496 (11) | 321/550 (58) |
| Site B | 30/93 (32) | 11/13 (85) | 46/645 (7) | 404/652 (62) |
| Site C | 27/90 (30) | 33/34 (97) | 35/552 (6) | 308/574 (54) |
| Site D | 15/52 (29) | 5/5 (100) | 17/509 (3) | 275/517 (53) |
| ***All sites*** | ***83/297 (28)*** | ***58/63 (92)*** | ***152/2202 (7)*** | ***1308/2293 (57)*** |
